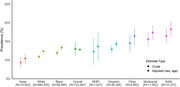# Prevalence of subjective cognitive decline among the US Multiracial population, 2019‐2023

**DOI:** 10.1002/alz70860_101323

**Published:** 2025-12-23

**Authors:** Tracy Lam‐Hine, Michelle C Odden, Bryan D James, David H Rehkopf

**Affiliations:** ^1^ Stanford University, Palo Alto, CA, USA; ^2^ Department of Internal Medicine Rush University Medical Center, Chicago, IL, USA; ^3^ Rush Alzheimer's Disease Center, Chicago, IL, USA

## Abstract

**Background:**

The prevalence of dementia and cognitive impairment in the growing US Multiracial (two or more race) population is unknown. Subjective cognitive decline (SCD), a self‐reported measure of worsening memory, is a proxy for dementia risk. We compared SCD prevalence nationally between Multiracial adults and other racial groups.

**Method:**

We analyzed Behavioral Risk Factor Surveillance System (BRFSS) data from 52 US states and territories (2019–2023) among adults aged 50+ identifying as White, Black, American Indian/Alaska Native (AIAN), Asian, Native Hawaiian/Pacific Islander (NHPI), other, Multiracial, or Hispanic. Weighted survey designs accounted for complex sampling, and Taylor linearization calculated standard errors for adjusted prevalence. Logistic regression adjusted for age and sex.

**Result:**

Among 723,497 participants, overall crude prevalence of SCD was 12.8% (95% CI: 12.6%–13.0%). Crude prevalence was highest for AIAN (18.2%, 95% CI: 16.2%–20.3%) and Multiracial adults (17.4%, 95% CI: 15.5%–19.3%). Other groups ranged from 10.4% (Asian, 95% CI: 8.9%–11.9%) to 13.7% (NHPI, 95% CI: 9.9%–17.5%). Overall age‐ and sex‐adjusted prevalence was 13.0% (95% CI: 11.1%–14.9%). AIAN adults had the highest adjusted prevalence (16.5%, 95% CI: 14.5%–18.4%), followed by Multiracial adults (15.6%, 95% CI: 13.7%–17.4%), Hispanic adults (13.0%, 95% CI: 11.9%–13.9%), and Black adults (11.9%, 95% CI: 11.2%–12.7%). Asian adults (9.4%, 95% CI: 8.0%–10.8%) and White adults 10.9% (95% CI: 10.4%–11.4%) had the lowest adjusted prevalence. Multiply imputed and complete case analyses had similar results.

**Conclusion:**

We provide the first estimates of SCD prevalence for the Multiracial population, revealing high rates comparable to AIAN adults. We could not disaggregate the Multiracial category using the publicly available BRFSS race data, a limitation. Future studies should examine cognitive impairment disparities in this group using clinical data and explore targeted preventive interventions.